# A portable graphite calorimeter for onsite reference dosimetry and beam quality correction factor determination

**DOI:** 10.1002/acm2.70381

**Published:** 2025-11-25

**Authors:** Nicolás Gómez‐Fernández, Jorge Deus‐Abreu, David Maughan, Faustino Gómez, Diego M. González‐Castaño

**Affiliations:** ^1^ Radiation Physics Laboratory, Area of Research Infrastructures University of Santiago de Compostela, E. San Lourenzo Santiago de Compostela Spain; ^2^ Dept de Ingeniería Mecánica, Máquinas y Motores Térmicos y Fluidos Universidade de Vigo, Campus Marcosende Vigo Spain; ^3^ National Physical Laboratory Teddington UK; ^4^ Departamento de Física de Partículas Universidade de Santiago de Compostela Santiago de Compostela Spain; ^5^ Fundación de Investigación Sanitaria de Santiago Santiago de Compostela Spain

**Keywords:** beam quality correction factors, calorimetry, reference dosimetry

## Abstract

**Purpose:**

To present a portable, reliable, and fast setup graphite calorimeter for onsite absolute dose measurements at hospitals.

**Methods:**

We have designed and built a portable graphite calorimeter optimized for fast setup and accurate measurements at clinical centers. Our system has minimum readout and auxiliary electronics and is inserted in a water fillable phantom in which chamber holders can be positioned in order to perform chamber calibrations at user's beam quality. System operation parameters were adjusted to obtain thermal equilibrium in minimum time. Our system accuracy was validated by inter‐comparison against the National Physical Laboratory (NPL) Primary Standard. Also, its portability and reliability have been tested during a measurement campaign in hospitals.

**Results:**

Optimization of system operation parameters together with an appropriate design yielded a fully portable, fast and easy‐to‐set‐up system with only 3 h treatment room occupation time. The system exhibits thermal stability of ± 100 µK and a maximum temperature drift of 60 µK/min, allowing for precise measurements with few repetitions. Mean relative local deviation between chambers calibrated by NPL calorimeter and our calorimeter was 0.1% demonstrating our system accuracy. We measured beam quality correction factors for four chamber types and performed a successful measurement campaign in five hospitals.

**Conclusions:**

We have developed an accurate absolute dosimeter that can be used for the determination of beam quality correction factors at clinical sites with minimal disruption of hospital workload.

## 1 INTRODUCTION

Reference dosimetry at clinical centres is currently based on a relative measurement approach: First, an ion chamber is calibrated in terms of absorbed dose to water at a reference beam at a Standars Dosimetry Laboratory (SDL) and then that chamber is used at the user site for measuring machine output under reference conditions. If a difference in beam quality exists between the calibration laboratory and the clinical centre, a beam quality correction factor ($k_{Q}$) has to be applied. Therefore, measurement at the clinical center is relative to measurement at SDL.

In this approach, each step (calibration at a laboratory reference beam quality, conversion of this calibration to user beam quality, and measurement at the clinical center) increases the overall uncertainty of reference dosimetry. Since every other relative measurement at the clinical center refers to the reference absorbed dose, its accurate determination is of concern in radiotherapy. Recommended accuracy in reference dosimetry is 2%^1^ therefore a measuring system with an uncertainty lower than this value will have a positive impact in radiotherapy.

A highly accurate method for measuring absorbed dose in water is through calorimetry, despite its poor spatial resolution, relative operation complexity, and longer measurement time compared to ionometric‐based dosimetry. Ideally, a portable calorimeter could be used at the clinical center to directly perform reference dosimetry by an absolute mean, or alternatively, a user chamber could be calibrated at the user beam quality against a portable calorimeter. Even if none of these options are possible, beam quality correction factors could be determined for the user chamber at several beam qualities against a calorimeter. TRS‐398 and others' current dosimetry protocols recognize the benefits of the latter option, as it would take into account individual chamber response variations.^2^, ^3^ Following this rationale, if a calorimeter can be used at a clinical center for beam quality correction factor determination of the user chamber at user beam qualities, individual response of the chamber for the exact user beam quality would be of benefit to the user, provided that the calorimeter accuracy has been properly validated. However, at a worldwide level, it has been traditionally impractical to use such systems in hospitals due to a lack of availability of commercial portable calorimeters^4^ and due to a lack of beam qualities matching that of clinical centers at calibration laboratories. Exceptions existed, notably NPL´s calorimeter system, which was used for determining experimental beam quality correction factors for a designated type of reference chamber and published in IPEM dosimetry protocol as early as 1990^5^. More recently Nederlandse Commissie voor Stralingdosimetrie has also published experimentally tabulated beam quality correction factors for a set of chambers in its dosimetry protocol.^6^ The last revision of the IAEA dosimetry protocol also incorporates experimentally determined data.^2^

A gradual introduction of various portable calorimetric systems has taken place over the past few decades, demonstrating the feasibility of using calorimetry in clinical beams.^7^, ^8^, ^9^ However, their widespread use is still limited, except for the initial commissioning of proton therapy centres, where it is a recommended practice.^10^ Thus, there is a growing need for these systems for the current expansion of proton therapy. Also, ultra high dose rate (UHDR) dosimetry research benefits from calorimetry as a very high dose rate is an advantage in this type of beam rather than an inconvenient.^11^

In this work, we present the description, characterization, and performance of a portable graphite calorimeter developed at the Radiation Physics Laboratory (RPL) and its initial experimental results in clinical beams. Aware of the heavy workload in hospitals, this calorimeter is specifically designed to minimally disrupt their clinical routine. The main features considered during the design process were: portability, quick use in the user's beam, and control of heat losses while keeping maximum dosimetric accuracy.

## 2 METHODS

### 2.1 System design

A portable graphite calorimeter with heat loss compensation was designed and assembled at the RPL. The calorimeter's design consists of four concentric high‐purity (99.9%) graphite cylinders separated by 1 mm air gaps and held together with wooden toothpick supports. The calorimeter is based on previous works^12^ and graphite cylinders are named as, from innermost to outermost: Core, jacket, shield, and medium. The set of graphite cylinders is supported by a polymethyl methacrylate (PMMA) structure. The core, in which absorbed dose is measured, has a diameter of 25.0 mm, a thickness of 2.3 mm, and a mass of 1.973 g. The external diameter of the medium cylinder is 80 mm.

Heat losses through air gaps between calorimeter layers can be decreased by using a vacuum pump.^13^ However, a very high vacuum is required in order to obtain a significant loss reduction.^14^ For system simplicity, portability, quick assembly, and fast setup, our system does not use a vacuum system, and therefore, the impact of heat losses was carefully evaluated to validate this approach (see section 2.3.4).

**FIGURE 1:**
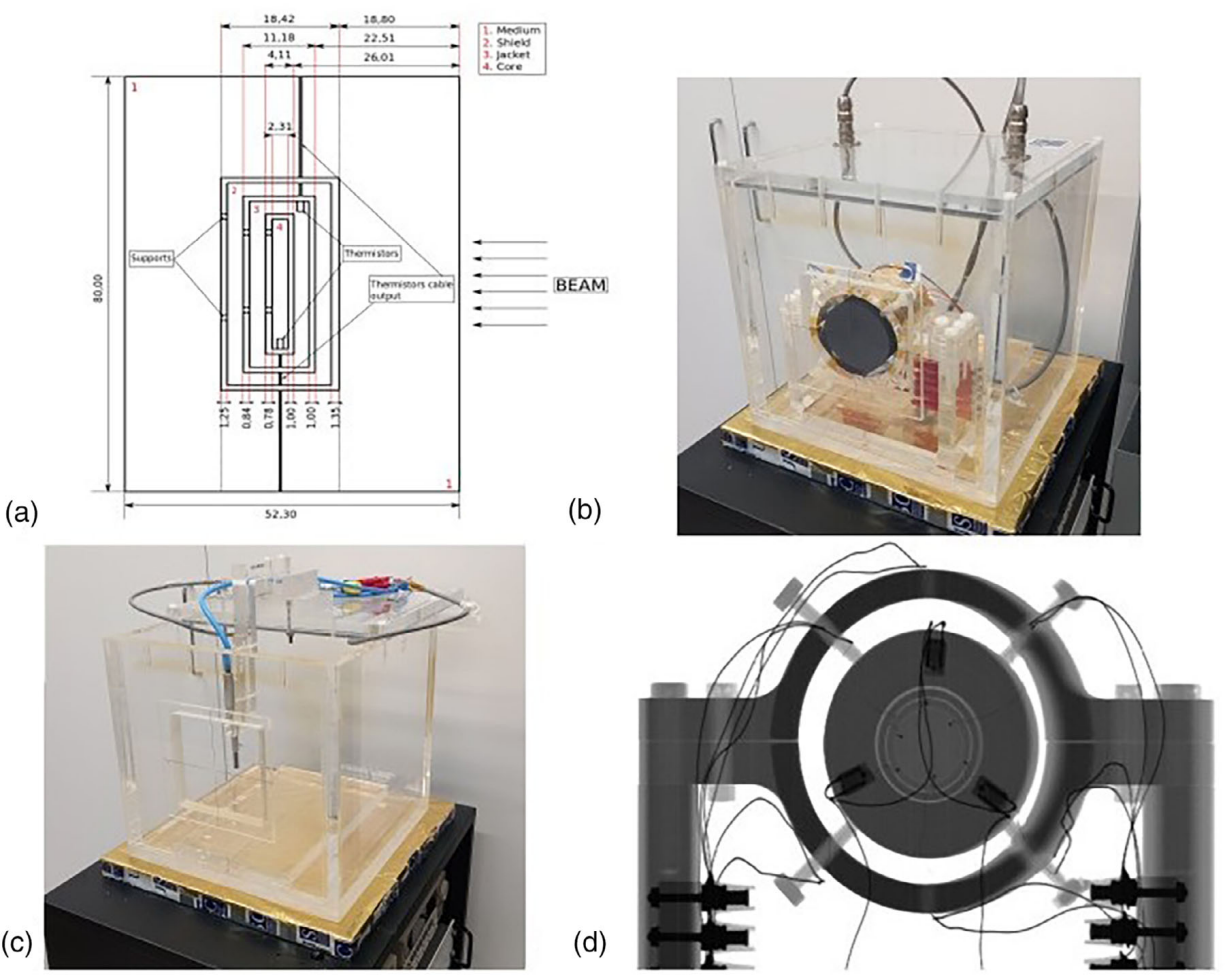
(a) Dimensions of graphite bodies. Beam field size is 10 × 10 cm. (b) Image of the calorimeter inside the empty tank without a polystyrene thermal shield. (c) Tank without calorimeter with ion chamber positioned for kQ determination or calibration. d) Radiograph of the calorimeter and its support.

Calorimeter and its support structure are placed inside a 330 mm side, PMMA walled container that isolates it from the external environment. The container is hermetically sealed with a removable PMMA lid. Additionally, 50 mm thick polystyrene panels and a layer of aluminized Mylar provide additional thermal insulation. The front wall of the container has a 5 mm‐thick PMMA entry window for horizontal radiation incidences. This window is covered only by aluminized Mylar, having no polystyrene covering it. Reference point of the calorimeter is located in the middle point of the core at a total mass thickness (including entrance window) of 5.0 gcm^−2^. This value was calculated by measuring each graphite part dimensions and mass, and PMMA entrance window thickness. Figure 1 shows the design and photographs of the system.

For performing chamber calibrations, the calorimeter can be easily removed, and the tank filled with water. Chamber holders can be attached to the lid in pre‐fixed positions that allow for precise chamber positioning at 5, 7, or 10 g cm^−2^ reference depths. This procedure ensures a chamber positioning repeatability below 0.2 mm, minimizing geometrical uncertainty in chamber calibrations.

The core has three 0.25 mm diameter TDK B57540G1 negative temperature coefficient thermistors embedded within it, with nominal resistance at 25°C of 10 kΩ. Two of these are part of a Wheatstone bridge for measuring temperature variations in the core, while the third can be used for dissipating electrical energy by acting as a heating resistor. The thermistors are attached to the core with epoxy resin, and the wires are coated with resin for electrical insulation. Each bridge has two thermistors connected to opposite legs, and two precision resistors of 10 kΩ are connected to the other two arms. The core bridge is connected to a Keithley 2181A nanovoltmeter (1 nV resolution at the most sensitive scale) and a precision current source, Keithley 6221, operating in delta mode^1^. Calibration of core thermistors in temperature is described in section i.c.i. Jacket has other three embedded thermistors of the same characteristics and functions. The jacket bridge is powered by an Analog Devices ADR421 low‐noise voltage regulator and connected to an Agilent 34410A multimeter. Shield has no thermistors.

Three 25 Ω power resistors of 5 W are embedded in the outer body of the calorimeter (medium), serving as heating elements for the calorimeter. The power dissipated by these resistors is controlled by a proportional‐integral‐derivative (PID) controller. This subsystem is used to bring the graphite bodies to a constant operating temperature by compensating for heat losses between the graphite bodies and the surrounding air.

Once the Wheatstone bridge is calibrated, the absorbed dose to graphite at the core​ $D_{g}$ is derived from the temperature increase Δ*T* induced by radiation within the core, using the following expression:

(1) $$D_{g}=c_{p,core}\cdot \Delta T\cdot k_{hl}\cdot k_{imp}$$

Where $c_{p,core}$ is the specific heat capacity of the core, including its non‐graphite components (thermistors, epoxy resin, and wooden supports), $k_{hl}$ is a correction factor for heat losses and is calculated using the finite element method (FEM), and $k_{imp}$ is a correction factor for core impurities and is calculated using:

(2) $$k_{imp}=\frac{m_{core}}{m_{g}+\sum_{i}m_{i}\frac{D_{i}}{D_{g}}}$$

Where $m_{core}$ is core mass, $m_{g}$ is the core mass of graphite in core, $m_{i}$ is impurities masses, $D_{i}$ is absorbed dose in core impurities, and $D_{g}$ is the absorbed dose in core graphite.^15^, ^16^ These doses are calculated with Monte Carlo simulation (see section 2.3.4).

In order to obtain absorbed dose to water, a conversion must be derived for each beam quality, in our case, through Monte Carlo simulation. Then Equation (1) becomes:

(3) $$D_{w}=c_{p,core}\cdot \Delta T\cdot k_{hl}\cdot k_{imp}\cdot \;C_{g,w}$$

Where $C_{g,w}$ is a general conversion factor for converting absorbed dose to graphite to the absorbed dose to water at the reference point. In our case, this factor includes conversion between absorbed dose to water and absorbed dose to graphite, but also corrections for air gaps, scatter differences due to the finite size of the graphite cylinder, volume averaging within the core due to non‐uniformity of the radiation beam profile, and position deviations. The accuracy of graphite to water conversion depends on the modeling of the radiation beam, the use of appropriate radiation transport (which includes algorithms and radiation‐matter interaction cross‐sections), and the careful modeling of geometries and composition of regions where the dose is scored. Simulations for obtaining $C_{g,w}$ corrections are explained in section i.c.iv..

### 2.2 System operation

A single National Instruments LabView script controls all electronic instruments associated with the calorimeter. This software enables the automation of temperature measurements in the core and jacket by setting optimal parameters for these measurements. It features a virtual PID controller, which uses core temperature as input and acts on the heating resistors to stabilize the temperature around 24.802°C, which is the real equilibrium point of the core Wheatstone bridge (determined by calibrating the bridge voltage drop in terms of temperature, as described in section i.c and looking for the temperature, which produces a null voltage). Maximum core temperature variation is ± 100 µK when the temperature is stabilized. Communication with the equipment is performed via a single Ethernet cable through an Ethernet switch.

Despite the relatively high specific heat capacity of graphite, the sensitivity of the system, in terms of temperature increase per unit of absorbed dose to graphite in the core, is about 1.2 mK/Gy. Given the range of absorbed dose to water rate produced in conventional radiotherapy beams, system temperature drift must be linear over a period at least thrice as large as the dose measurement. Additionally, we consider a maximum slope of 2 µK/s over 60 s before starting a measurement. This is of particular concern if a portable system is to be used in clinical locations, where temperature control is sometimes centralized by the hospital's maintenance services, and significant variations may occur.

The calorimeter described in this work operates in quasi‐adiabatic mode.^13^ Thus, potential temperature drift not corrected by the PID during irradiation must be corrected. We have accounted for the temperature drift by performing a temperature versus time linear fit using data recorded before and after irradiation during time intervals with a minimum duration of half the irradiation time which itself lasts between 20 and 40 s. These linear fits are then extrapolated to the midpoint of the irradiation time interval to calculate the temperature variation, ΔT, between these linear regressions. This approach corrects for non‐perfect thermal stability reached by the PID and is valid whenever heat transfer between bodies remains constant in the considered time intervals of the pre‐ and post‐irradiation linear fits. All measurements taken are recorded in an ASCII text file for later analysis. Resultant temperature variation is converted to absorbed dose to water using Equation (3).

### 2.3 System characterization

#### 2.3.1 Calibration of Wheatstone bridges

Calibration of the core Wheatstone bridge in temperature was carried out before full system assembly in a separate experiment. First, the core and its three thermistors were enclosed in a waterproof latex envelope and submerged in a thermal bath, itself inside an adjustable temperature oven. A PT100 probe connected to a calibrated telethermometer was also immersed in the bath to measure reference water temperature, in contact with the calorimeter core. The oven temperature was set to various values, allowing each selected temperature to stabilize for several hours. When the temperature is stable (variations about 10 mK in 1 min) 10 min period, the temperature and output voltage of the Wheatstone bridge were measured every 10 s. The core bridge was calibrated between 24.30 and 25.02°C.

#### 2.3.2 Determination of the specific heat capacity

**FIGURE 2:**
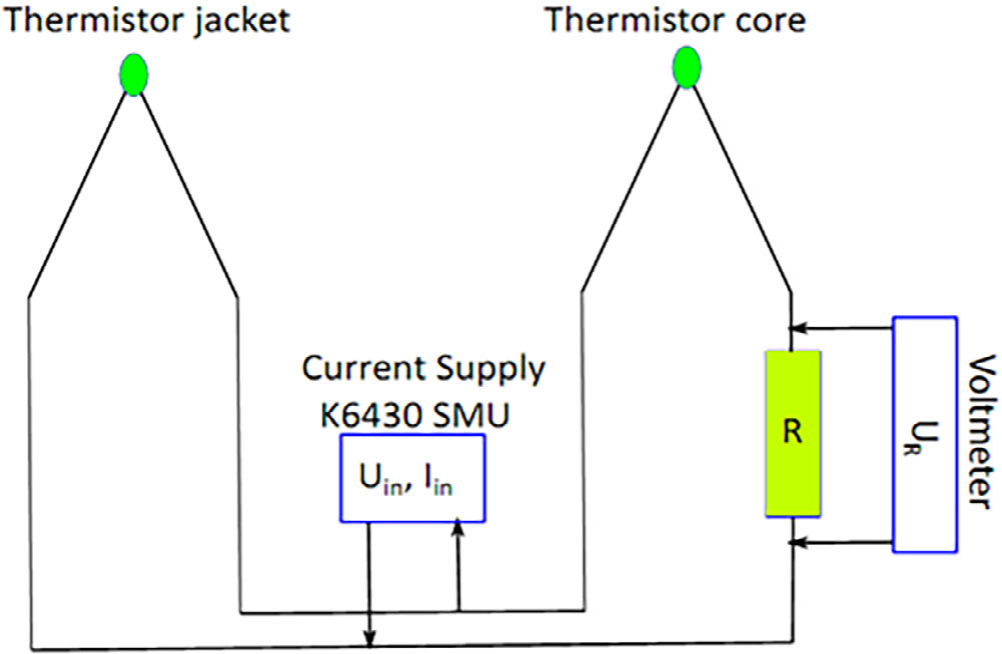
Circuit diagram for measuring the specific heat capacity of the core. Core and Jacket thermistors are connected in parallel. Using the Keithley 6430 SMU, a known amount of current is supplied, and the input voltage is measured. Agilent 34410A is used to measure the voltage drop across *R*. *R* also allows limiting the core thermistor current, balancing the power dissipated in core and jacket to obtain an equal temperature increase and minimize thermal losses.

Once the full calorimeter was assembled, the specific heat capacity of the core and thermistors assembly was determined with the setup shown in Figure 2. A high‐precision^2^ Keithley 6430 calibrated source measurement unit was used to supply a known electrical power to the heating thermistors in the jacket and core. Through the use of a variable resistor R, the circuit is balanced to ensure that the power dissipated in the core and jacket raises the temperature of both bodies by the same amount and at the same rate. Since we know the current supplied by the current source (I_IN_), as well as its input voltage (U_IN_), by measuring the voltage drop across a resistor (U_R_) in the circuit using an Agilent 34410A multimeter, we can determine the power dissipated by each thermistor. This allows us, once the Wheatstone bridge of the core is calibrated in temperature, to calculate the specific heat capacity of the core and even enables the possibility of operating the system in a quasi‐isothermal mode. Core specific heat capacity is calculated as:

(4) $$E=P\cdot t=c_{p,core}\cdot m_{core}\cdot \Delta T$$

Where E and P are the total thermal energy and power dissipated in the calorimeter core, calculated as $\;\left(U_{in}-U_{R}\right)\cdot \left(\frac{U_{R}}{R}\right)$, *t* is the time during which power is dissipated, $m_{core}$ is the mass of the core, ΔT is the change in temperature of the core measured with the calibrated Wheatstone bridge, and $c_{p,core}$ ​is the specific heat capacity of the core, including its non‐graphite components (thermistors, epoxy resin, and wooden supports).

A linear regression fit of energy versus temperature increase was performed to calculate $\;c_{p,core}$. Different values of electrical energy were supplied to the core. The procedure followed involved applying several well‐known currents through the heating thermistors for a known time. As a consistency test, a constant current was maintained and the time during which the current flowed was varied through the circuit and thermistors, yielding to same result.

In spite of using point heating for calibration, given the high thermal conductivity of graphite, no significant biases on measurements of radiation‐induced‐quasi‐uniform heating distributions will be produced, as has been shown in previous works.^17^

#### 2.3.3 Evaluation of heat losses

Our system uses a PID connected to heating resistances embedded in the outermost body to bring all bodies to a constant temperature above ambient temperature. After warming up, in the initial state, all calorimeter bodies are close to equilibrium, but small temperature drifts exist. When irradiation starts, the dose is not delivered at exactly the same rate to all parts of the calorimeter, and therefore temperature differences will exist, which will induce heat transfer between bodies. In order to mitigate this, the core is placed at such a depth in graphite as to be in the attenuation region of the photon PDD curve, and therefore higher dose delivered to the top layer of the jacket will be compensated (in terms of temperature) by the lower dose delivered to the bottom layer of the jacket. Also, lateral thermal disequilibria will be minimized by irradiating a field with enough lateral extension. Even in this situation, since the system does not have an ideal behavior, there can be small heat losses from core to jacket when the system is irradiated.

A detailed three‐dimensional heat exchange numerical model of the calorimeter was implemented in Ansys Fluent 2022 R2 to study these losses. A three‐dimensional mesh of 283854 tetrahedral volumes was created, with sizes between 1 and 2 mm^3^. Special care was taken to have a thin mesh in the air gaps to properly capture heat exchange between bodies. Air was assumed to be still. Thermistors, wires, and epoxy were not considered in the simulation geometry. Several initial tests were performed to evaluate the differences in heat exchange between considering still or moving air. These tests showed that heat flux due to convection was less than 1% of heat flux due to conduction. This finding agrees with the fact that the Rayleigh number of the confined flow is of the order of 1.12E‐04, which is below the natural convection critical value.^18^ This result is consistent with the low dimensions of the air gap combined with the low temperature differences. The effect of thermistors and the thermal control system was introduced into the numerical model by starting from thermal equilibrium and imposing a fixed external temperature boundary condition in the outermost layer of the device. The heat conduction equation was solved with the finite volume method (FVM) using a second‐order upwind scheme for the spatial discretization and a first‐order implicit scheme for the time discretization. A time step of 0.1 s was imposed, which is below the time scale of the solid heat conduction problem. A volumetric heat source was imposed in each graphite body depending on the beam dose distribution. The values of power dissipated in the different bodies of the calorimeter were calculated using a Monte Carlo radiation transport simulation for various MV beam field sizes: 10 × 10 cm, 5 × 5 cm, 4 × 4 cm, 3 × 3 cm, and 2 × 2 cm. By integrating heat losses over a period equal to irradiation time, we calculated heat loss correction $k_{hl}$ as a function of field size. We considered several irradiation times to find an optimal irradiation time for each measurement. Results obtained were validated by comparing ion chamber‐measured output factors with heat loss corrected calorimeter‐measured values.

#### 2.3.4 Monte Carlo simulations

In this work, we used the EGSnrc code.^19^ Several radiation units were modeled, and these models were used to generate phase space files representing reference fields, with BEAMnrc code.^20^ The units considered were a cobalt‐60 AECL Theratron unit (typical standard calibration quality), a Varian CLINAC DHX, a Varian TrueBeam, and Elekta Versa. Some of the models were previously developed and validated in other works.^21^, ^22^ In the present work, the same modeling methodology was used to create reference fields for Elekta Versa 6FFF, 6 MV, and 10 MV beam modalities. In addition to 10 × 10 cm reference fields^3^, smaller fields were also simulated for heat loss studies. All phase spaces are recorded at reference distances ranging between 93 and 100 cm, according to user reference conditions, for LINAC beams. Additional phase spaces for cobalt‐60 are recorded at 80, 70, and 60 cm for dose rate range experiments. For every particular LINAC measured with the calorimeter, its simulation model was validated by comparing locally measured and simulated dose distributions.

These phase space files were used as radiation source for two complementary radiation transport simulations using egs_chamber code^23^:

- First one calculated absorbed dose at a reference point located at the same mass thickness as the calorimeter core $D_{w}$, using the same PMMA‐walled tank described in section i.a. filled with water. Absorbed dose to water was scored at a 2 × 2  × 1 mm voxel.
- Second one involves a detailed model of our calorimeter (including its PMMA support, air gaps, density variation between bodies, etc.) set inside the same PMMA‐walled tank as described in section i.a. In this simulation, we scored absorbed dose to graphite in the calorimeter core, $D_{g,cal}$.

Monte Carlo conversion factor for each phase space representing a reference field is calculated as follows:

(5) $$C_{g,w}=\frac{D_{w}}{D_{g,cal}}$$

For radiation beams producing a non‐homogeneous fluence across the calorimeter core (such as FFF beams), the averaging effect is taken into account by the Monte Carlo simulation of absorbed dose to water to a smaller voxel. On the other hand, the lack of scatter in the calorimeter measurements for radiation fields wider than the graphite external diameter (8 cm) is also accounted for in the simulation.

Additionally, for heat loss studies, varying side square field phase spaces were used as a source in dosrznrc^24^ simulations in order to determine absorbed dose distributions in the different graphite bodies and calculate power dissipated in each body in the aforementioned heat loss numerical simulations.

$\frac{D_{i}}{D_{g}}$ ratios from Equation (2) were also determined through simulations in order to calculate $k_{imp}$. This ratio was computed for each non‐graphite material (thermistors, adhesive, and supports) by simulating the calorimeter body with the impurity masses present in it. The volumes of the impurities were estimated based on the measurements taken, and the atomic composition was determined from the component datasheet. Simulations were made for three different energies: Cobalt‐60, 6, and 15 MV.

#### 2.3.5 System performance

Temperature stability affects the minimum measurable dose rate, similarly to how signal‐to‐noise ratio affects any detector measuring an electrical signal. Therefore, we investigated the relationship between dose rate and accuracy. To do this, we measured absorbed dose rate in water delivered by one of the cobalt‐60 irradiators at RPL (the one with a lower activity source) as a function of source to surface distance, while keeping the field size constant at any given distance, and compared the result with that of a secondary standard ionization chamber. We evaluated repeatability as a function of dose rate, adding to the previous measurements others conducted with photon beams from the RPL electron linear accelerator. Also, reproducibility was evaluated by repeating absorbed dose to water measurements in cobalt 60 and 6 MV reference beams after complete disassembly and re‐assembly over a period of several days.

Core temperature sampling frequency (which acts as input to the PID), PID parameters, and action period during different phases (heat up, pre‐irradiation, and irradiation) were optimized for maximum thermal stability, minimum heat up time, minimum heat loss, and maximum overall system accuracy.

Portability of the system and room occupation times were tested in a measurement campaign in which the output of 5 LINACS in five different nearby hospitals was determined. We added RPL own LINAC. In this campaign, we transported our system to these centers and measured absorbed dose to water for all available beam qualities at reference conditions. We compared our results with those obtained by users with their local means and procedures. Every local ionometric standard used in the campaign, including RPL, had traceability to the Physikalisch‐Technische Bundesanstalt (PTB) Primary Standard in Braunschweig (Germany). Before and after all shipments, the system was scanned by x‐rays.

Absorbed dose to water measurements performed with the graphite calorimeter (D_w,Q_)_CAL_ and the hospital measurements carried out with the ionization chamber (D_w,Q_)_IC_ at each beam quality Q were compared using the normalized error ratio

(6) $$E_{n}=\frac{\left|{\left(D_{w,Q}\right)}_{CAL}-{\left(D_{w,Q}\right)}_{IC}\right|}{\sqrt{U{{\left(D_{w,Q}\right)}_{CAL}}^{2}+U{{\left(D_{w,Q}\right)}_{IC}}^{2}}}\leq 1$$

considering the existence of an agreement if the normalized error ratio, $E_{n}$, was smaller than or equal to 1.

#### 2.3.6 Uncertainty budget

Different sources were considered in the evaluation of the uncertainty of absorbed dose to water determined through Equation (3). Type A and B components were considered for each factor. Type B uncertainties for $c_{p,core}$ and ΔT include components associated with the calibration and resolution of instruments used for their determination. For tabulated values used in $c_{p,core}$ determination, published data on their uncertainty were considered. Monte Carlo simulation type B uncertainty in $C_{g,w}$ calculation arises from the modeling of the LINAC and calorimeter geometry and materials, and LINAC primary source free parameters. The heat loss correction factor has only type B uncertainty associated with the limited capacity of the model when reproducing experimental heat losses.

Calibration coefficients determined with our system have additional uncertainty components related to output reproducibility between calorimeter and chamber measurements, as well as uncertainty in chamber positioning with respect to calorimeter reference point, chamber readout, saturation effect determination, and a component associated with electrometer correction factor.

Beam quality correction factors determined through equation (7) have an uncertainty lower than twice that of the calibration coefficients since some factors do not apply, such as output reproducibility in cobalt 60 quality or electrometer correction, which cancels out.

All reported uncertainties are shown with a coverage factor *k* = 1

#### 2.3.7 Validation of dosimetric accuracy

Accuracy of the system was validated through a bilateral inter‐comparison traceable to the National Physical Laboratory (UK) Primary Standard of absorbed dose for photon beams^4^. In this exercise, six chambers from both institutions (Two NPL 2611, one NE 2571, one PTW T30013, and two PTW T30012‐1) were calibrated against each participant's respective calorimeter in several flattened beam qualities (TPR_20,10_ of 0.667, 0.688, 0.735, 0.760) available at their laboratories and at nearby hospitals, including cobalt 60 reference beam quality (0.563). Since not all of beam qualities matched exactly, interpolation was required in order to compare calibration coefficients at equal beam qualities. A comparison protocol was agreed beforehand, considering the existence of agreement if normalized error ratio, $E_{n}$, calculated between calibration coefficients for a given chamber and beam quality Q, $N_{D,w,Q}$, was smaller or equal to 1:

(7) $$E_{n}=\frac{\left|{\left(N_{D,w,Q}\right)}_{RPL}-{\left(N_{D,w,Q}\right)}_{NPL}\right|}{\sqrt{U{{\left(N_{D,w,Q}\right)}_{RPL}}^{2}+U{{\left(N_{D,w,Q}\right)}_{NPL}}^{2}}}\leq 1$$

Where subindex RPL or NPL denotes values determined at each participant's laboratory.

Beam quality correction factors were derived from the calibration coefficients of the previous section as the ratio of calibration coefficients shown in Equation (8), since in both laboratories, cobalt 60 reference beams were available.

(8) $$k_{Q}=\frac{N_{D,w,Q}}{N_{D,w,Q_{0}}}$$

Additionally, beam quality correction factors were measured for five more chambers (three PTW T30013, one T30006, and one IBA FC65‐G) in flattened (TPR_20,10_ of 0.667, 0.688, 0.735, 0.741, 0.760) and unflattened (TPR_20,10_ of 0.632, 0.677, 0.705) beam qualities. Experimentally derived kQ factors were compared with recently published data.

## 3 RESULTS

### 3.1 System characterization

#### 3.1.1 Wheatstone bridge calibration

**FIGURE 3:**
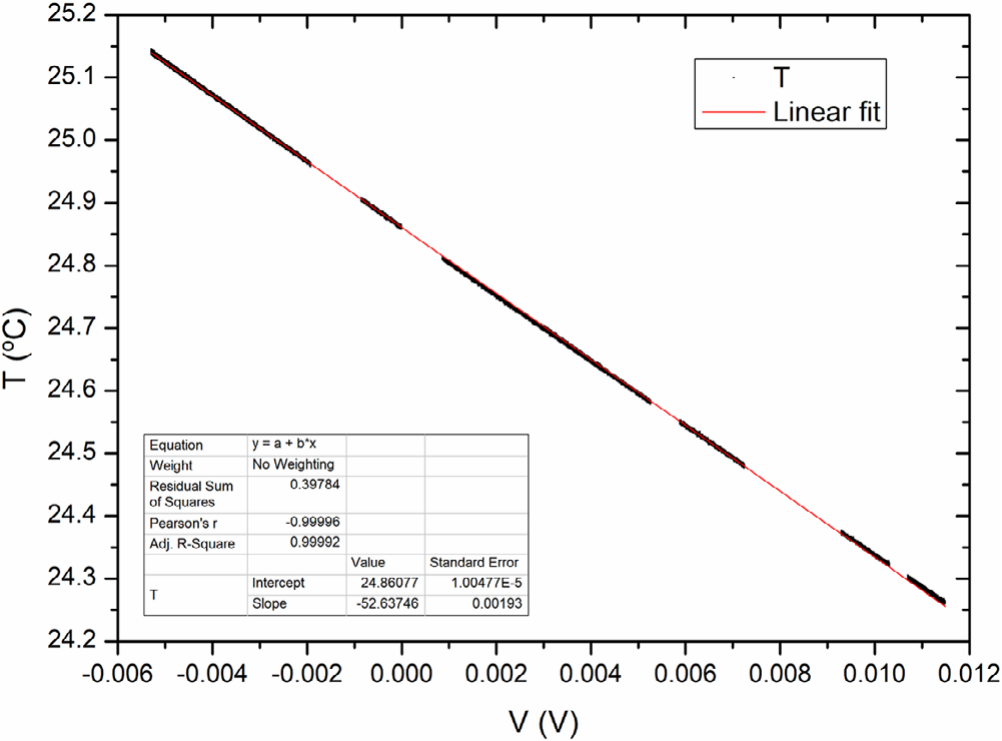
Linear fit of core Wheatsone bridge voltage drop versus thermal bath temperature used for bridge calibration.

Wheatstone bridges were calibrated in terms of temperature in the range from 24.30 to 25.02°C. Operating temperature is extracted from the calibration curve and corresponds to a voltage drop of 0 V. Since operating temperature is 24.802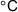, this temperature interval would allow for measuring up to a maximum absorbed dose of 180 Gy, before needing to reset operating temperature by switching off PID heating. Temperature presents a standard deviation of 2 × 10^−3^°C for each measurement of bridge output voltage. Result of the linear fit (shown in Figure 3) for the core is: 

(9)

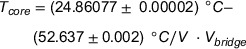




#### 3.1.2 Specific heat capacity

**FIGURE 4:**
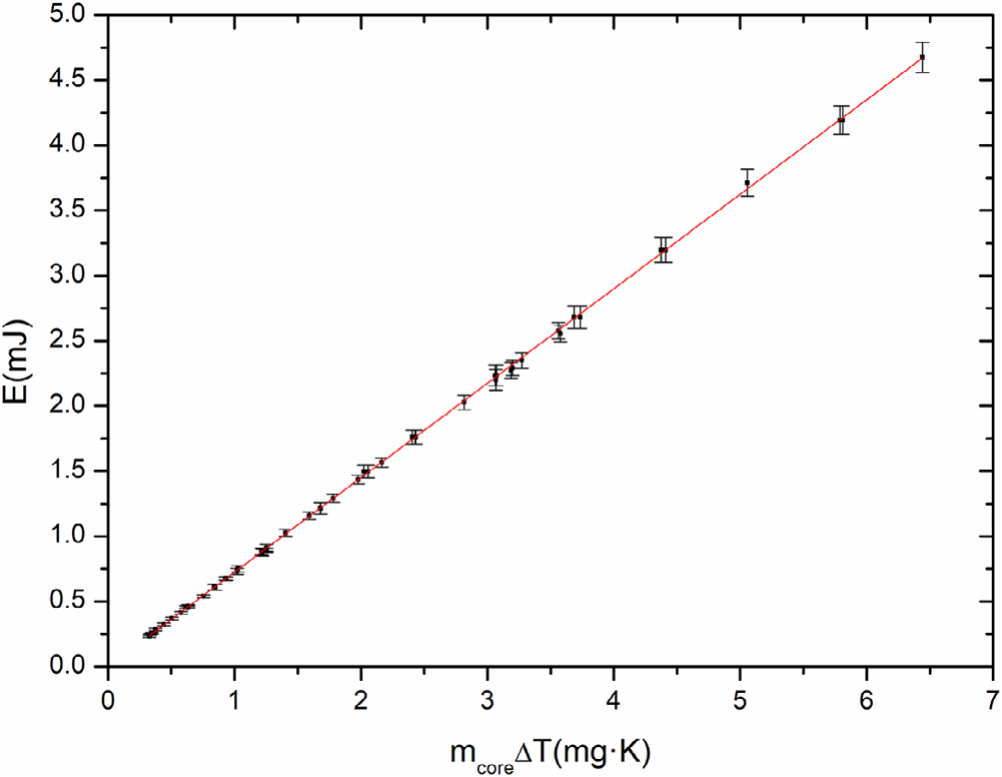
Linear regression between dissipated thermal energy and temperature change in the calorimeter core. The fit parameters were used to calculate the core specific heat capacity.

Specific heat capacity was measured at a range of temperatures regulated by the PID, which included the optimal calorimeter working temperature of 24.802°C. Figure 4 shows the measured temperature increase as a function of dissipated thermal energy to core and jacket. If we use the slope of the linear fit of dissipated power versus temperature increment in Equation (4) and we use specific heat capacities and masses of non‐graphite core components detailed in Table 3, we can calculate a specific heat capacity value of:

$$c_{p,core}=\left(72.0\pm 1.5\right)\;\frac{J}{\mathrm{kg}\cdot \;K}$$

#### 3.1.3 Heat losses

Detailed simulations confirmed that the largest contribution to the heat losses from the core is due to convection caused by the movement of air between the core and the jacket. For a dose rate of 6 Gy/min, heat losses (k_hl_) as a function of field size relative to an ideal situation in which the core will have no losses are 0.15%, 0.23%, 0.42%, 2.58%, and 11.83% for 10 × 10 cm, 5 × 5 cm, 4 × 4 cm, 3 × 3 cm, and 2  × 2 cm, respectively. A minimum filed size can be determined by establishing a heat loss limit of 0.5%, leading to 4 cm x 4 cm. This field size would produce homogeneous heat dissipation within the core and jacket. For the largest field size, where absorbed doses to core and jacket (and therefore respective temperature increases) are closer, heat loss correction is minimal. However, as irradiation time increases, the small temperature difference increases and therefore losses increase in a non‐linear way, leading to unacceptable levels. In order to avoid this, irradiation time must be kept below 40 s. With such measurement times, heat losses for a field size wider than 5 × 5 cm are kept below 0.2% and can be corrected. We use a correction factor $k_{hl}$ of 1.0015 for measurements under reference conditions (field size 10  × 10 cm).

#### 3.1.4 Monte Carlo calculated correction factors

Monte Carlo conversion factors for several beam qualities and for Source to Surface Distance of 100 cm and a field size of 10  × 10 cm defined at source to surface distance are shown in Table 1. For other SSDs and field sizes considered in this work, values may differ slightly.

**TABLE 1:** General conversion factors from absorbed dose to graphite core to absorbed dose to water, calculated for different radiation beam qualities, both with flattening filter (WFF) and flattening filter free (FFF).

Radiation source	Beam quality (TPR20,10)^5^	General conversion factor (C_g,w_)
**Cobalt 60 irradiator**	0.563	1.064 ± 0.002
**Beam qualities with flattening filter (WFF)**		
**Varian CLINAC DHX / varian true beam 6 MV**	0.667	1.067 ± 0.002
**Elekta versa / elekta precise 6 MV**	0.688	1.069 ± 0.002
**Elekta versa / elekta precise 10 MV**	0.735	1.073 ± 0.002
**Varian CLINAC DHX / varian true beam 10 MV**	0.741	1.073 ± 0.002
**Varian CLINAC DHX / varian true beam 15 MV**	0.760	1.078 ± 0.002
**Beam qualities without flattening filter (FFF)**		
**Varian true beam 6 FFF**	0.632	1.074 ± 0.002
**Elekta versa 6 FFF**	0.677	1.072 ± 0.002
**Varian true beam 10 FFF**	0.706	1.080 ± 0.002

Results obtained for k_imp_ were 0.9994 ± 0.0007 (TPR_20,10 _= 0.563), 0.9994 ± 0.0007 (TPR_20,10 _= 0.667), and 0.9993 ± 0.0007 (TPR_20,10 _= 0.760).

#### 3.1.5 System performance

For optimal performance, the PID input temperature is sampled every 0.1 s while the action period of the PID controller (the interval between changes in power transferred to the heating resistor) is 5 s. During irradiation, PID is inactive for 60 s (20 s pre‐irradiation, 20 s irradiation, and 20 s post‐irradiation), returning to 5 s once the irradiation is complete. The PID target temperature is updated to the core temperature value after irradiation. In this configuration, the maximum time to reach thermal equilibrium (time to reach a state where the maximum core temperature variation is ±100 µK) is around 90 min (depending on initial temperature). In this state, results from repeatability and reproducibility, in terms of standard deviation, are shown in Table 2 as a function of dose rate and for two radiation beams. The number of repetitions needed to achieve each standard deviation is also shown as a measure of the time needed to perform a precise measurement. Short‐term LINAC output repeatability was evaluated with an ionization chamber secondary standard in a separate experiment and was found to be 0.03%. Irradiation time for cobalt 60 was 30 s, and the number of Monitor Units delivered at a dose rate of 600 MU/min for the LINAC was 200.

**TABLE 2:** Repeatability and reproducibility of measurements performed with the graphite calorimeter in radiation beams of the Radiation Physics Laboratory.

Radiation field	Dose rate (Gy/min)	Number of repetitions	Repeatability (%)	Number of repetitions	Reproducibility (%)
**Cobalt 60 reference beam**	0.196	25	0.77	3	0.34
	0.328	23	0.46	2	0.41
	0.435	9	0.32	2	0.42
	0.598	14	0.16	3	0.30
**6 MV reference beam**	6	9	0.03	10	0.10

**FIGURE 5:**
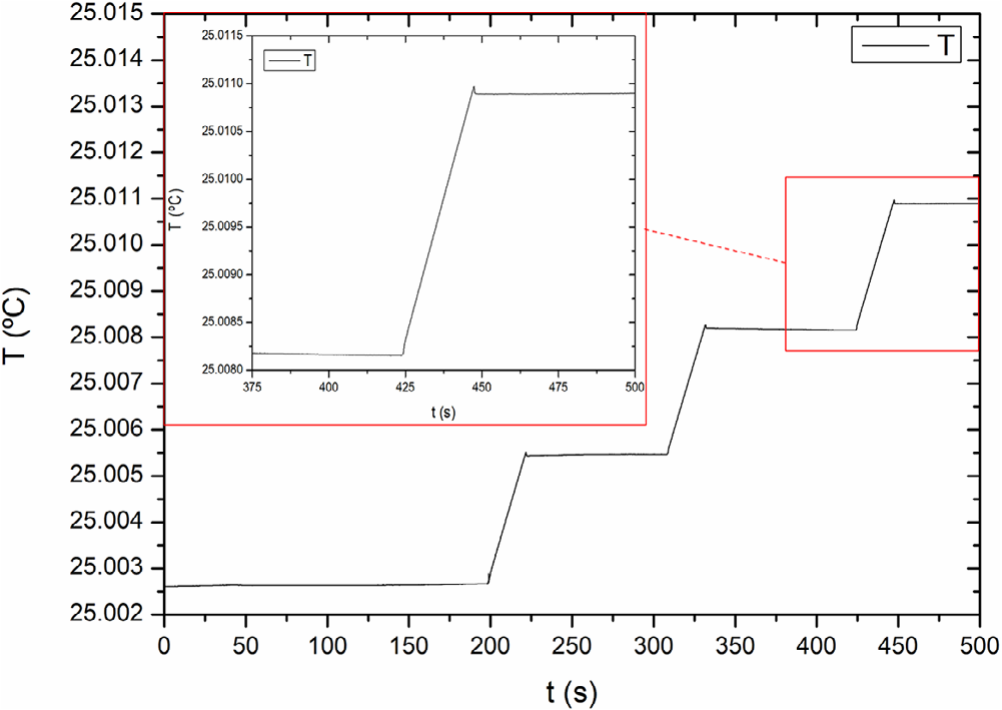
Graphite core temperature versus time for a series of three consecutive 200 MU irradiations at a hospital centre. It is observed that the PID is capable of correcting any drift in the system's temperature. A single run is enlarged for more detail.

Figure 5 shows a typical result of a measurement carried out at a clinical center under the conditions indicated above. Three irradiations of 20 s are observed. After an irradiation, a waiting period of about 100 s is allowed for the PID to adjust and stabilize around the new temperature reached, if necessary. After this series of irradiations, which corresponds to an absorbed dose in water of approximately 10 Gy, we can observe that the temperature in the core remains stable. Thermal drift remains within 1 µK/s over 60 s and exhibits a steady behavior.

**FIGURE 6:**
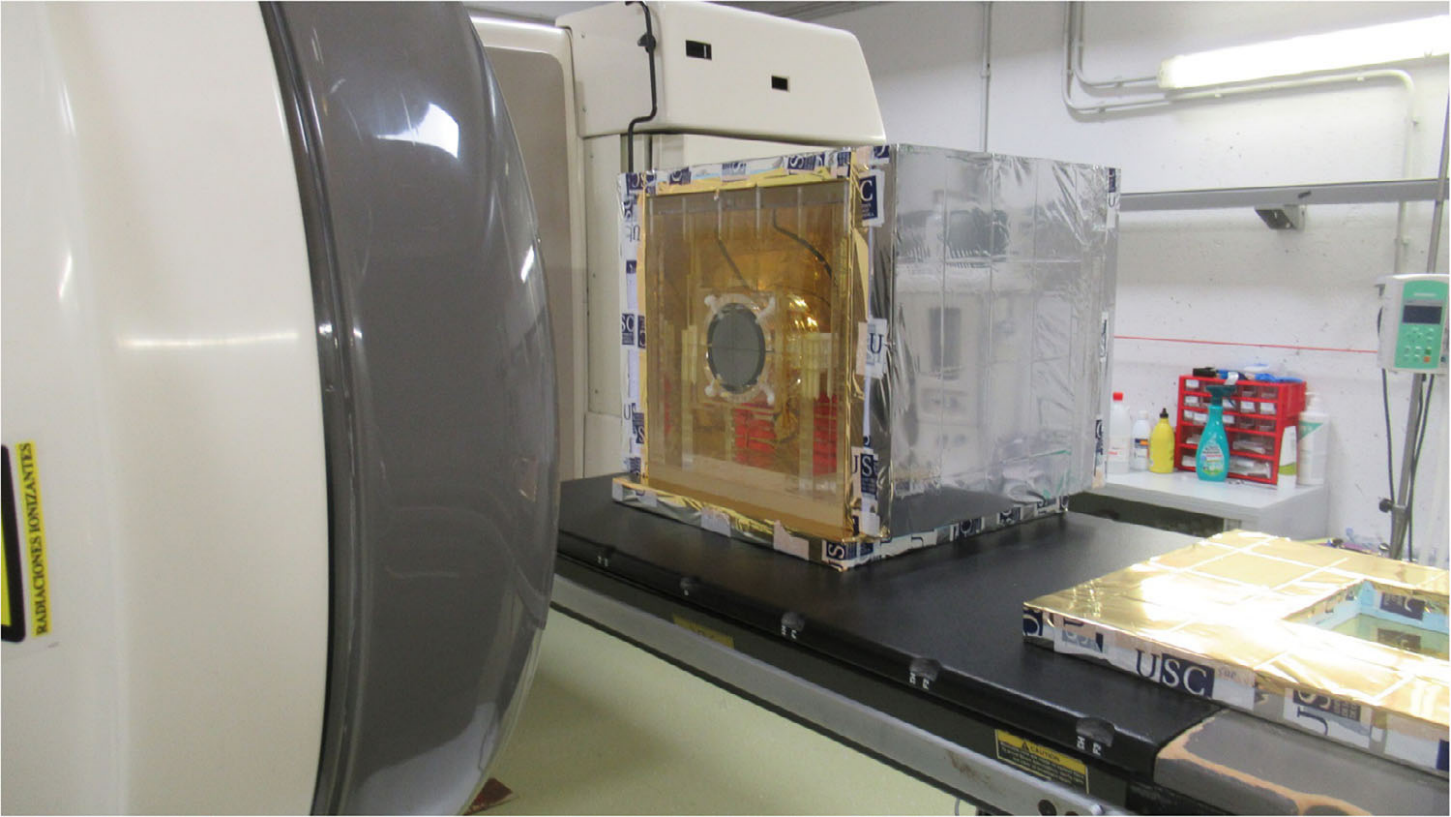
Calorimeter setup in a radiotherapy room at a hospital during a validation campaign.

**FIGURE 7:**
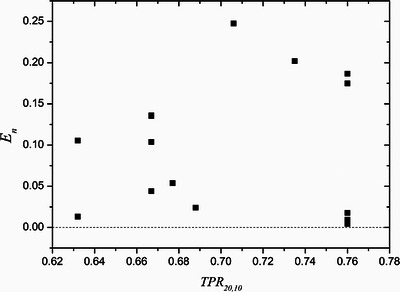
Normalized error ratio between hospital equipment (ionization chamber and electrometer) and graphite calorimeter. Uncertainties are 2% and 1%, respectively.

An image of the whole system in a hospital during the validation campaign is shown in Figure 6. Total treatment room occupation time for a reference dosimetry audit session measuring three beam qualities was less than 160 min, including setup and heat up (90 min), dose measurements (45 min), and de‐assembly (20 min). If a user chamber calibration is performed in three beam qualities, then the measuring campaign takes up to 245 min^6^. Figure 7 shows normalized error ratios between measurements of absorbed dose to water with our calorimeter and with user dosimetry equipment as a function of beam quality.

No damage was observed in the system in any of the x‐ray images during the campaign.

#### 3.1.6 Uncertainty budget

Uncertainty sources and overall standard uncertainty for absorbed dose to water determination under reference conditions for a clinical photon beam are shown in Table 3, and for calibration of an ion chamber at the same user beam in Table 4.

**TABLE 3:** Uncertainty components for absorbed dose to water for a clinical photon beam.

Component	Type A	Type B
**Specific heat capacity**	0.13	0.15
**Impurities correction**		0.06
**Monte Carlo conversion factor**	0.05	0.25
**Heat loss correction**		0.15
**Temperature readout**	0.03	0.17
**Cuadratic sum**	0.14	0.37
**Combined standard uncertainty**	0.40	

**TABLE 4:** Uncertainty components for calibration of an ion chamber in user beam at a hospital center.

Component	Type A	Type B
**Dose to water**		0.40
**Positioning**		0.15
**Chamber Reading**	0.05	0.10
**Electrometer calibration**		0.15
**Beam reproducibility**	0.10	
**Saturation effect**		0.20
**Quadratic sum**	0.10	0.48
**Combined standard uncertainty**	0.52	

Combined standard uncertainty assigned to kQ determination is 0.65%.

#### 3.1.7 Dosimetric accuracy

**FIGURE 8:**
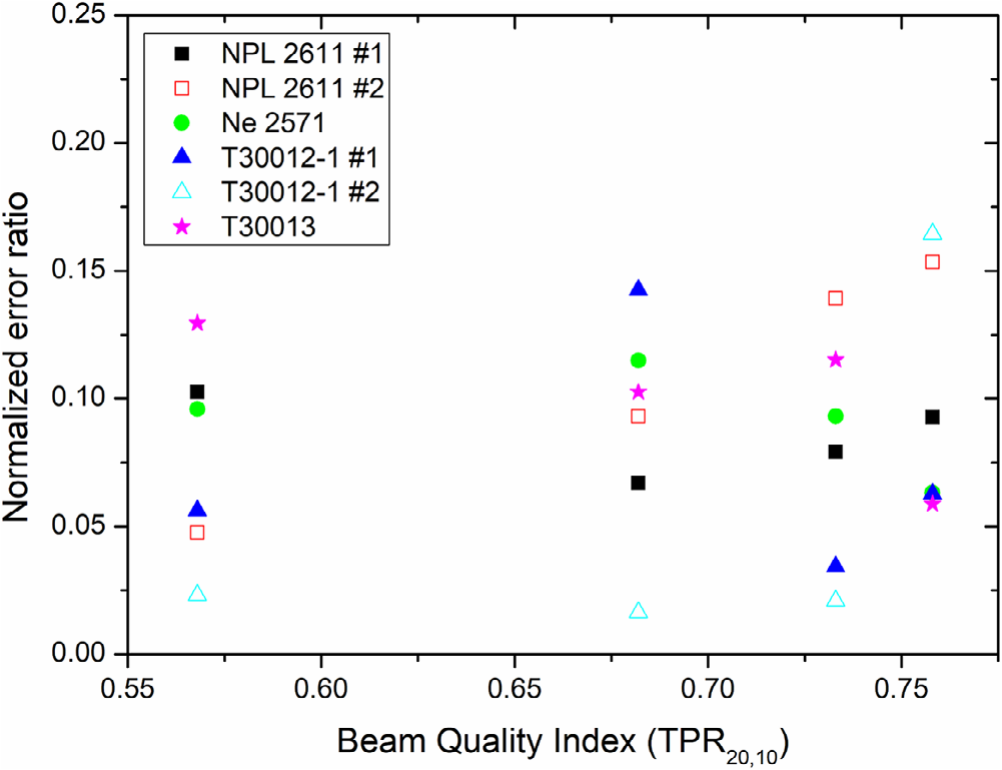
Normalized error ratios calculated between calibration coefficients obtained from the graphite calorimeter at the National Physical Laboratory (UK) and the Radiation Physics Laboratory (Spain) for six chambers and four beam qualities. Uncertainties are 1.4% and 1%, respectively.

Results of the inter‐comparison are shown in Figure 8 as normalized error ratios between calibration coefficients for the six chambers considered. All results agreed according to the predefined pass criterion. Reported uncertainties were 1.3% for NPL results and 1% for RPL results, with a coverage factor of *k* = 2. Minimum, maximum, and mean normalized errors were 0.02, 0.16, and 0.09, respectively. These correspond to minimum, maximum, and mean absolute values of relative local differences of 0.03%, 0.27%, and 0.14%. Mean of signed relative local differences is 0.05%.

**FIGURE 9:**
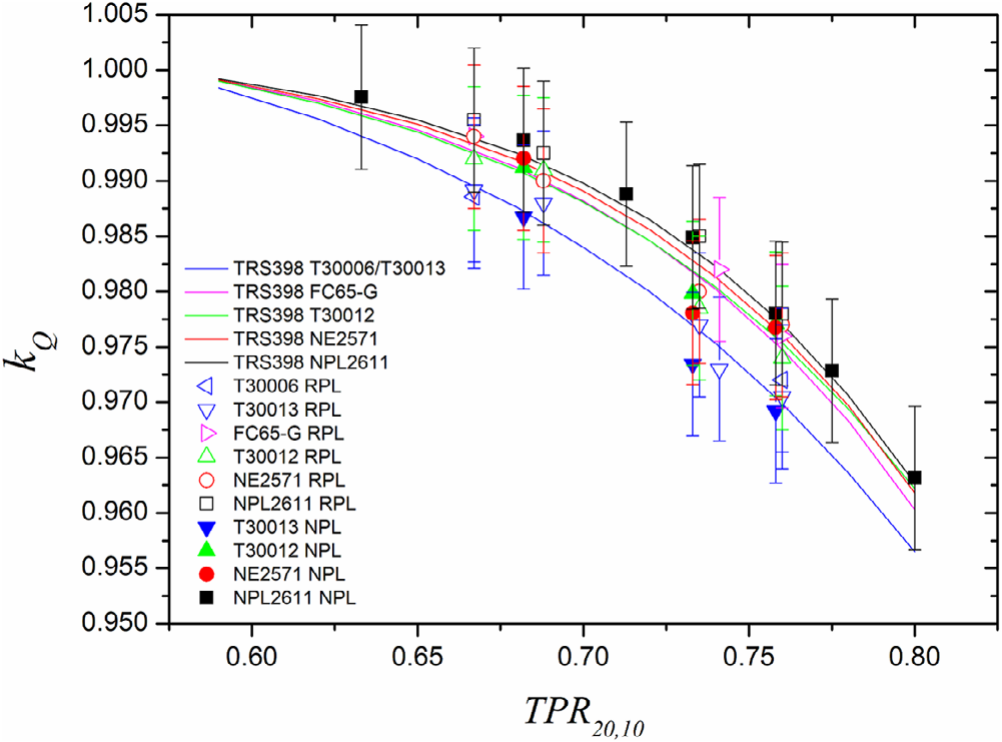
Beam quality correction factors as a function of beam quality index (TPR_20,10_) measured by the National Physical Laboratory (UK) and Radiation Physics Laboratory (Spain) for six chamber types. Suggested TRS398 rev1 values are also included in the plot.

**FIGURE 10:**
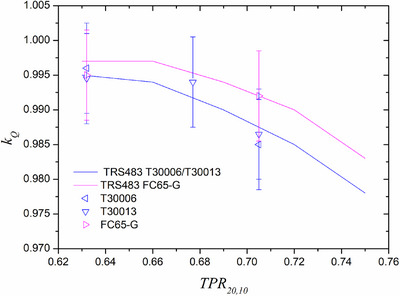
Beam quality correction factors as a function of beam quality index (TPR_20,10_) measured by Radiation Physics Laboratory (Spain) for three chamber types. Suggested TRS483 values are also included in the plot.

Beam quality correction factors measured by both participant laboratories for chamber types considered in the bilateral inter‐comparison are shown in Figure 9 (with flattening filter) and Figure 10 (without flattening filter), together with additional results measured at hospitals. For chamber models for which there was more than one unit, the results shown are averaged. For a given laboratory, the maximum relative difference between units of the same model was 0.3%. The maximum relative local difference between both laboratories for a given chamber was 0.4%. The maximum relative local difference between any experimental result and TRS‐398 or TRS‐483 was 0.3% for the RPL data.

## 4 DISCUSSION

Active temperature control implemented in our system allows achieving highly accurate dose measurements while keeping room occupation time at a minimum and regardless of hospital central climate control of the irradiation room. Therefore, our system design fulfils its main goals.

The calorimeter achieved better thermal equilibrium more quickly by using core temperature as input for PID. By receiving input from the innermost and most thermally isolated body and actuating over the less isolated body and by selecting an appropriate refresh interval, the PID behavior is less prone to big jumps in thermal injection. For better results, it is necessary for the thermal drift to remain as constant as possible during irradiation. Therefore, the PID maintains thermal injection at a constant level while the system is being irradiated. This approach produces good results as long as irradiations do not last more than 40 s.

Although the bridge reference voltage is lower than that used in other systems in the literature, the power dissipated in the core and jacket is not negligible compared to the power dissipated by radiation. Also, the power dissipated in the core and jacket is not perfectly balanced. However, PID can bring the system very close to thermal equilibrium, by compensating net heat losses, resultant temperature fluctuations, and drift the system to detect dose rates as small as 0.2 Gy/min and even measure with acceptable accuracy dose rates of 0.4 Gy/min. Dose rates of 0.6 Gy/min can be measured with 0.1% repeatability by increasing the number of repetitions over 30, which is acceptable in terms of room occupation time in an SDL. For LINAC dose rates of 6 Gy/min, readout repeatability is 0.03% for nine repetitions, as good as the readout repeatability of an ion chamber.

Special care has been made to validate heat losses through air gaps, since a vacuum is not used to increase thermal insulation between layers. This choice helps keep the system simpler and easier to transport, set up, and operate, which was a main design goal. Whenever the field size is above 5  × 5 cm, heat losses can be kept at reasonable levels. System performance shows that for the reference field size, heat losses are almost negligible, as predicted by numerical simulations, and therefore confirm the feasibility of using air as an insulator rather than vacuum.

Monte Carlo conversion factors are similar to others found in literature,^13^, ^25^ although different, as other systems are built in a different geometry.

All results from the inter‐comparison exercise showed agreement within 0.3% (0.1% on average); therefore, our system is compatible with the National Physical Laboratory primary standard, even in the inter‐comparison conducted in the radiation beams of clinical centers, a fact that proves its accuracy in absorbed dose to water determination even at non‐laboratory conditions and with a minimum treatment room occupation time.

Beam quality correction factors determined experimentally with the calorimeter are similar to those recently published in the new revision of TRS398, which considers a fit of MC and experimental data. This means that our system can be used to extend data for other chambers and beam qualities.

The system accumulates at the moment of this writing 5000 km of transport by road and 3000 km by plane without any malfunction, so its portability has been demonstrated. During the measurement campaign treatment, room occupation times were kept below 3 h, which is an affordable time for a quality control activity, which will not be scheduled in less than two‐year intervals. It is worth mentioning that the results of the hospital dosimetric audit campaign, included in this work, showed a normalized error ratio below 0.25 for all beam qualities, indicating good agreement between the measurements with the graphite calorimeter and the ionization chambers ± . This is similar to or better than what is published in the literature for other dosimetry audit exercises, but since calorimetry is a more accurate method than alanine, TLD, or ionometry, these results give more confidence to the user.

## 5 CONCLUSIONS

We have developed a portable graphite calorimeter for use in laboratory and clinical environments. The system can be used in a clinical environment with maximum accuracy while keeping treatment room occupation time to a minimum. The main reasons for this are the excellent thermal stability acquired with the optimally configured PID controller and the small heat losses obtained in short irradiation times and wide enough radiation fields. The thermal stability provided by the PID control loop and the relatively low power dissipated by the measuring Wheatstone bridges allow the system to measure µK temperatures, avoiding the use of a vacuum.

Inter‐comparison calibration results performed with our system both in the laboratory and in the clinic environment showed differences lower than 0.3% respect to the NPL primary standard. The device was used in a reference dosimetry audit campaign in five hospitals with a minimum occupation time and with an uncertainty in absorbed reference dose of 0.8%. All users’ results were compatible with the calorimeter within uncertainties. We also obtained megavoltage beam quality correction factors with an uncertainty of 1% for reference type chambers compatible within ± 0.2% with other experimentally derived data published in dosimetry protocols.

## AUTHOR CONTRIBUTION

*Designed, assembled, and characterized the calorimeter*: Nicolás Gómez‐Fernández. *Carried out the experimental campaigns at RPL and at hospitals, and made the data analysis*: Nicolás Gómez‐Fernández and Diego M González‐Castaño. *Performed the numerical simulations for heat losses*: Jorge Deus‐Abreu. *Calibrated the chambers at NPL for the inter‐comparison*: David Maughan. *Reviewed the manuscript*: Faustino Gómez. *Coordinated the Calorimeter development project, performed the Monte Carlo Simulations for conversion factors*: Diego M González‐Castaño. All authors contributed to the discussion of results.

## CONFLICT OF INTEREST STATEMENT

The authors declare no conflicts of interest.

## Notes

1 Delta mode triggers the current source to alternate the signal polarity, and then triggers a nanovoltmeter reading at each polarity. This technique cancels out any thermoelectric offsets (constant and drift over time) and the results reflect the true value of the voltage.

2 1 nA at 100 uA range, 1 uV at 200 mV range.

3 Defined at a distance from source of 100 cm

4 At NPL the user chamber is calibrated in terms of absorbed dose to water by direct comparison with NPL reference chambers (that have been directly compared to the NPL calorimeter) in a water phantom, using at least three beams from the NPL Elekta linac and NPL ^60^Co beam.

5 TPR_20,10_ for cobalt 60 units was measured at a Source to Chamber Distance (SCD) of 100 cm and for a field size of 10 cm x 10 cm defined at SCD. For FFF fields, TPR_20,10_ was measured at SCD = 100 cm and an indicated field of 10 cm x 10 cm.

6 A full characterization of recombination effect would require measuring the Jaffe plot and that could take several extra hours. However, that part can be carried out by the user by its means in a separate experiment at reference conditions.
